# A Division of the Symphisis Pubis, Attended with Compleat Success in a Case of Difficult Parturition

**Published:** 1803-01-01

**Authors:** 

**Affiliations:** Surgeon of Saint Mihiel


*A Division of the Symphisis Pubis, attended with compleat Success in a Case of difficult Parturition, by* Citizen Manscey, *Surgeon of Saint Mihiel; communicated by our Correspondent at Paris.*

THE subject of this operation, who was the wife of a farmer of the department of the Meuse, 36 years of age, of a small stature, and whose sister had already been delivered of two children, piece meal, by means of the crotchet. This was the second time that this woman had been pregnant. The first of which, she was delivered by a surgeon after several days labour; but the child, by the avowal of persons present, was considerably smaller than in the present case.

Cit. Manscey was called 30 hours after the commencement of the pain; he found the woman suffering and nearly exhausted; the pains were yet strong and bearing down; the contractions of the matrix were sensible to the touch, on laying the hand on the abdomen; but the head, which was in the first position, did not stir, and the pains were diminishing. The external labia and adjacent parts were much swollen, which induced the author to conclude that the head was engaged. The parts were in high inflammation, and exquisitely sensible; the umbilical cord hung out to the length of six or seven inches, and had the appearance of lung, yet in a sound state. The summit of the head presented a tumour, hard and elastic, from which the author inferred that the child still lived, but it was impossible to introduce the finger between the head and pelvis except at the extremities of its transverse diameter. The parietal bones were so closely pressed between the pubis and sacrum, that it was impossible to suppose the existence of any part of the brain between them; all efforts to return the head were fruitless, and the author conceived that every circumstance warranted him to conclude the head locked in the pelvis, though it was a case which he knew occurred but seldom, and much less frequent than was supposed some years ago. Believing the child alive, the author first endeavoured to introduce the forceps, which he did separately through the spaces at the extremities of the transverse diameter; but every effort to carry them between the head, sacrum and pubis, proved ineffectual, as well as every trial to move the head by approaching the jaws of the forceps in the direction in which they were introduced. The patient, fatigued by these repeated trials, required rest, and the author left her for some hours.

At four o'clock in the morning of the 17th Thermidor, no progress whatever had been made; the pains became weaker, and the patient lost all courage, so as to cry out to have her belly opened. The author was without assistance or advice; the Cæsarean operation, which he was at liberty to perform, was rendered inadmissible from the high state of inflammation, the excessive efforts and fatigued state of the uterus, and the belief of the head being locked, locked, so as even to render it very difficult to withdraw it through an opening in the abdomen. This operation would have been performed by the author if he had visited the patient at an earlier period; but under every circumstance of the case, he decided on the division of the symphisis, the probable success of which was increased by the very particular position of the head.

The operation was done in the following manner, which the author thinks it necessary to detail, as no description is given in any work on the operation of surgery: [*The description is however to be found.*]

The patient was placed on her back, her loins a little elevated, the legs and thighs bent, as in the operation for the stone; the particular position of the bed, obliged the operator to place himself between the thighs. The integuments were kept tense by the index finger and thumb, and with a bistoury he divided the integuments and fat, to about two inches and a half; the opening of one of the external pudical arteries, produced considerable hæmorrhage, owing to the great determination of blood to these parts in this state of the womb; this, as well as some other branches divided in the course of the operation, was stopped by ligature; the symphisis was next divided by carrying the incision from above downwards, first by cutting through two-thirds of its thickness, and then insinuating the finger behind the symphisis so as to direct the bistoury, which, with a second incision, separated the cartilage entirely. The symphisis divided, the author attempted to separate the bones, which he found impossible to do, owing to many ligamentous fasicculi, which lay behind, being undivided. This being done, a gradual separation was effected by pressing on the elevations of the ilia; this was strongly seconded by the pains, which became strong, so that in a short time he could introduce the thumb and index finger, and which nearly equalled two inches. A strong pain soon succeeding, the head descended and protruded at the os externum, and after a quarter of an hour the entire body was extracted, lifeless, and the after birth was followed but by a small quantity of blood.

The author follows up the description of the operation by a detail of the subsequent treatment, in which we find nothing remarkable. The wound was dressed simply, and the edges were brought together by means of a bandage surrounding the pelvis. The fourth day after the operation, the patient was attacked by an inflammatory complaint of the lungs, accompanied by spitting of blood, but without any fixed pain; this gave way to the antiphlogistic treatment after some days.

On the 26th day after the operation, an inflammation and suppuration appeared under the glutei muscles of the right thigh; and being opened, it was found to descend to the junction of the ileum and sacrum; the suppuration was abundant, but diminished, and was perfectly healed, at the end of a month from the operation. The patient complained during the treatment of a numbness in the extremity on one side, but was soon able to walk with the assistance of a stick, and at length engage in her ordinary occupations. She complains however of an inability to retain her urine (a consequence sometimes of the application of the forceps or of difficult labours), but on dry days and fine weather can contain for a time her urine. Owing to ulceration which had occurred during the cure, some partial adhesions had taken place in the vagina. The author concludes, by justifying his prefering this operation to the Cæsarean, by reasons drawn from the circumstances of this particular case, and which have been already stated, and endeavours to remove some objections advanced against the operation, such as the difficulty of reuniting cartilaginous surfaces, &c. without however declaring himself a decided partizan for the operation.

